# Kinesiological Analysis Using Inertial Sensor Systems: Methodological Framework and Clinical Applications in Pathological Gait

**DOI:** 10.3390/s25144435

**Published:** 2025-07-16

**Authors:** Danelina Emilova Vacheva, Atanas Kostadinov Drumev

**Affiliations:** Department of Physical Medicine, Rehabilitation, Occupational Therapy and Sports, Medical University—Pleven, 1, Saint Kliment Ohridski Street, 5800 Pleven, Bulgaria

**Keywords:** inertial measurement units (IMUs), pelvic oscillation, gait analysis, hip joint, recovery

## Abstract

**Highlights:**

**What are the main findings?**

• Inertial sensors detected significant pelvic oscillation abnormalities in elderly hip surgery patients, especially in the frontal and transverse planes.
• Pelvic control improved significantly over time, particularly when walking without assistive devices, although values remained below normal.

**What is the implication of the main finding?**

• Wearable inertial measurement units provide sensitive, objective gait monitoring beyond traditional clinical observation.
• This technology enables tailored rehabilitation strategies that enhance functional recovery in orthopedic patients.

**Abstract:**

Accurate gait assessment is essential for managing pathological locomotion, especially in elderly patients recovering from hip joint surgeries. Inertial measurement units (IMUs) provide real-time, objective data in clinical settings. This study examined pelvic oscillations in sagittal, frontal, and transverse planes using a wearable IMU system in two groups: Group A (n = 15, osteosynthesis metallica) and Group B (n = 34, arthroplasty), all over age 65. Gait analysis was conducted during assisted and unassisted walking. In the frontal plane, both groups showed statistically significant improvements: Group A from 46.4% to 75.2% (*p* = 0.001) and Group B from 52.6% to 72.2% (*p* = 0.001), reflecting enhanced lateral stability. In the transverse plane, Group A improved significantly from 47.7% to 80.2% (*p* = 0.001), while Group B showed a non-significant increase from 73.0% to 80.5% (*p* = 0.068). Sagittal plane changes were not statistically significant (Group A: 68.8% to 71.1%, *p* = 0.313; Group B: 76.4% to 69.1%, *p* = 0.065). These improvements correspond to better pelvic symmetry and postural control, which are critical for a safe and stable gait. Improvements were more pronounced during unassisted walking, indicating better pelvic control. These results confirm the clinical utility of IMUs in capturing subtle gait asymmetries and monitoring recovery progress. The findings support their use in tailoring rehabilitation strategies, particularly for enhancing frontal and transverse pelvic stability in elderly orthopedic patients.

## 1. Introduction

The ability of living organisms to move through their environment is fundamental to survival—a process scientifically referred to as locomotion. In humans, walking represents the primary and most natural form of locomotion. Walking is a highly coordinated motor activity that enables the body to navigate through physical space. Early humans depended on this function to gather resources and transport them to favorable living environments [1]. Later studies established that upright posture and bipedal locomotion evolved primarily to optimize energy efficiency during movement. Walking is considered automated motor behavior, characterized by economical muscle activation patterns and efficient mechanical movement. This efficiency is primarily maintained by the ventral inertial momentum of the body’s center of mass (COM) [2].

In recent years, the analysis of the human gait has garnered significant scientific attention. Research in this domain aims to provide quantitative and objective measurements of gait parameters, with applications spanning sports science, rehabilitation medicine, geriatrics, and even forensic identification. From a kinesiological standpoint, gait is defined as the translational displacement of the body through the environment, achieved via coordinated rotational movements of the joints [3]. The entire musculoskeletal system is engaged, with the lower limbs performing the majority of the motor activity [4]. Notably, human gait is highly individualized—often referred to as a biomechanical “fingerprint.” Gait abnormalities are most frequently observed in individuals with neurological disorders affecting the central or peripheral motor neurons. These impairments disrupt muscle tone, balance, proprioception, and coordination, and manifest in characteristic ways across different pathologies. For instance, post-stroke hemiparesis often presents with a Wernicke-Mann gait; multiple sclerosis is associated with spastic gait; and Parkinson’s disease leads to the hallmark shuffling, festinating gait. Spastic diplegic gait is commonly seen in cerebral palsy (Morbus Little), while spinal cord injuries result in paraplegic gait patterns. Peripheral nerve damage can produce a flaccid or steppage gait. When the cerebellum or vestibular system is affected—as in tumors, inner ear disorders, or cerebellar degeneration—ataxic gait patterns emerge. In hemiataxia, where only one cerebellar hemisphere is impaired, patients may veer toward the affected side. A distinctive example is the spastic-ataxic gait in multiple sclerosis, indicating combined pyramidal and cerebellar involvement. These patients often exhibit stiff, slow movements with extended lower limbs, and many eventually lose ambulatory function. Tabetic gait, caused by neurosyphilis or dorsal column dysfunction, is marked by a loss of proprioception and awareness of limb positioning. Among ataxic patterns, the so-called staggering gait resembles that of an intoxicated individual, with exaggerated lateral deviations and corrective swaying. Astasia-abasia, seen in frontal lobe lesions, features a paradoxical inability to stand or walk despite intact supine motor function. Psychogenic disorders can result in a hysterical gait, where patients simulate impairments in the absence of actual deficits. The Parkinsonian gait is notable for static tremors, bradykinesia, rigidity, reduced arm swing, and forward-leaning posture. Affected individuals move with short, shuffling steps, often propelled by inertia, and are at an increased risk of falling. Traumatic injuries of the lower extremities also significantly compromise gait. These include fractures of the femur, knee, tibia, and ankle; ligamentous injuries requiring reconstructive surgery; and joint replacements due to osteoarthritis. In addition, systemic diseases such as rheumatoid arthritis, diabetes, and certain cardiovascular or respiratory conditions can reduce functional independence. Age-related degenerative changes also contribute to gait decline in the elderly, representing a substantial portion of the population with locomotor limitations. Accurate and objective analysis of gait parameters is essential for early diagnosis, monitoring of disease progression, prevention of complications, and the development of individualized treatment plans [5].

In clinical and research contexts, gait analysis methods are broadly categorized into traditional and modern techniques. Traditional gait assessment relies on observational analysis. Clinicians evaluate a patient’s gait through visual inspection, ideally without the patient’s awareness, to infer the underlying cause, whether neurological or orthopedic. While practical and widely used, this approach is inherently semi-subjective. In contrast, modern technologies have enabled a more precise and objective measurement of gait parameters [6]. Despite the advances in non-wearable sensor systems, their high cost, technical complexity, and lack of portability limit widespread clinical use. This highlights the need for wearable sensor technologies that offer objective, reliable, and accessible gait assessment in real-world settings, enabling continuous monitoring and individualized rehabilitation.

Contemporary gait analysis devices fall into three main categories: non-wearable sensor (NWS) systems, wearable sensor (WS) systems, and hybrid systems that combine elements of both. Non-wearable sensor systems are designed for use in controlled laboratory environments, where fixed sensors collect data as subjects walk along predefined paths [7]. These systems are divided into two main types: image-processing (IP) systems and floor-sensor (FS) systems. IP systems employ digital or analog cameras, often using infrared, laser range scanning (LRS) or time-of-flight (ToF) technologies to capture motion. Through techniques such as pixel filtering, thresholding, and background subtraction, they enable real-time analysis of gait via depth and distance measurements. FS systems use pressure-sensitive platforms embedded in the floor to measure ground reaction forces and pressure distribution [6,7]. These platforms record the magnitude and timing of forces applied during the stance phase, offering granular insight into the biomechanics of gait. Advanced FS systems feature high-resolution sensor matrices, capable of differentiating pressure across discrete regions of the foot. NWS systems are lauded for their high accuracy and reliability [8]. However, several barriers limit their routine clinical application. These include substantial cost, complex technical setup, and spatial demands that necessitate dedicated laboratory infrastructure. Installation and calibration often require skilled technicians, increasing time and resource expenditure. Moreover, their non-portable nature renders them impractical for real-time monitoring in naturalistic or home environments—particularly for patients with mobility limitations. Despite these limitations, the detailed biomechanical data provided by NWS systems make them indispensable in high-precision research and diagnostic settings [9].

Wearable sensor systems represent a transformative innovation in gait analysis by facilitating data collection during everyday activities [10]. These portable devices incorporate various sensors such as accelerometers, gyroscopes, magnetometers, goniometers, ultrasound transducers, and electromyography (EMG) sensors, strategically placed on the body (e.g., feet, shanks, hips, or lower back) to measure physiological and biomechanical parameters [11]. Inertial measurement units (IMUs), which integrate accelerometers, gyroscopes, and sometimes magnetometers are among the most widely adopted tools for gait analysis [6,7,11]. Accelerometers capture linear acceleration, while gyroscopes detect angular velocity and rotational displacement [12]. When fused, these components enable detailed 3D motion tracking, supporting the calculation of parameters such as step length, stride frequency, cadence and symmetry [13]. Goniometers, often built on strain gauge technology, measure joint angles during dynamic movement [14]. Ultrasound sensors can supplement spatial data acquisition by assessing parameters like step width and stride length [15]. Surface electromyography (EMG) provides valuable insight into muscle activation patterns by detecting the electrical activity generated by muscles during contraction. EMG is particularly useful for evaluating neuromuscular coordination, fatigue, and the timing of muscle recruitment during gait [16]. However, like goniometry and ultrasound, EMG typically requires controlled laboratory environments, fixed setups, and skilled personnel for accurate data acquisition and interpretation [6,14,15,16]. These constraints limit their applicability in real-world clinical settings or during routine rehabilitation assessments.

**Figure 1:**
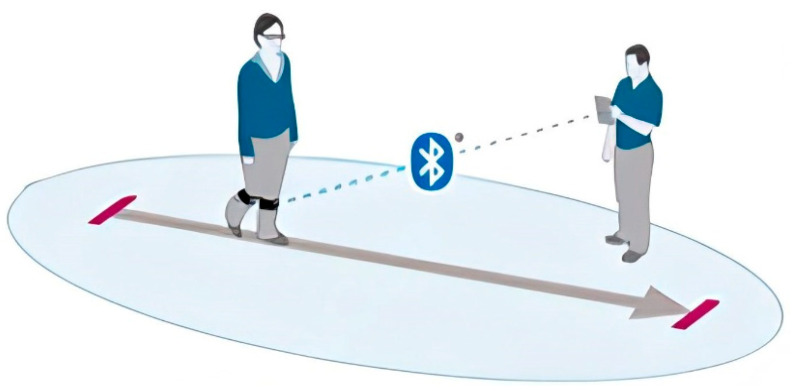
Illustrative visualization of gait analysis with WS technology.

In contrast, wearable sensors provide a portable, accessible, and cost-effective alternative for gait and balance assessment [17]. These systems, commonly incorporating IMUs, include accelerometers, gyroscopes, and magnetometers that allow for three-dimensional motion capture in real-world environments. When strategically placed on anatomical landmarks such as the pelvis or lower limbs, WS enables continuous monitoring of dynamic activities without restricting natural movement (see Figure 1) [18].

The adoption of WS in clinical settings has expanded rapidly due to their ability to collect spatiotemporal gait parameters such as cadence, step length, gait speed, and stance-to-swing phase ratios. Additionally, parameters like pelvic symmetry, stride variability, and gait stability can also be derived, offering valuable insights into functional performance and recovery progression [19]. The principal advantages of WS include minimal setup time, ease of use, and the possibility for frequent, ecologically valid assessments. These features make WS especially useful for tracking functional improvements during rehabilitation in individuals with lower limb injuries, degenerative diseases, or post-surgical recovery. By enabling objective measurement and longitudinal monitoring, WS support data-driven clinical decisions and personalized therapeutic interventions [20]. Nevertheless, certain limitations remain. Measurement accuracy may be affected by sensor drift, soft tissue motion artifacts, or external interference, and the interpretation of the resulting data still requires clinical expertise in gait biomechanics. Despite these challenges, the role of WS in contemporary rehabilitation continues to grow, reinforcing their value as an essential tool for evaluating functional mobility and guiding recovery strategies [21].

Recent advances in inertial sensor technology have markedly enhanced the accuracy and reliability of gait analysis. Modern systems now integrate multiple sensors with sophisticated algorithms, allowing for more precise motion tracking and data interpretation. These innovations have expanded the role of inertial sensors in personalized medicine by enabling tailored rehabilitation programs and offering predictive insights into functional recovery [22]. Since independent walking is often impaired in individuals with lower limb trauma or pathology resulting in reduced mobility, social isolation, and diminished quality of life—there is an increasing clinical demand for accessible and objective tools to assess and monitor gait abnormalities [23]. This study was motivated by the need to establish a practical, clinically applicable methodology for gait assessment using wearable inertial sensor systems.

Despite advances in gait analysis technology, there remains a need for practical, objective assessment tools that can be easily implemented in routine clinical rehabilitation, particularly for elderly patients with differing surgical treatments. Understanding pelvic oscillation across multiple planes provides critical insights into balance and mobility recovery, which are essential for designing personalized rehabilitation strategies.

Drawing on existing literature and clinical observations, we hypothesize that elderly patients undergoing rehabilitation after hip surgery will exhibit significant improvements in pelvic oscillation across the sagittal, frontal, and transverse planes, as measured by wearable inertial sensor systems. Furthermore, we expect that patients who have undergone arthroplasty will exhibit greater improvements in pelvic motion parameters compared to those with osteosynthesis metallica, reflecting differences in surgical intervention and recovery dynamics.

The aim is to develop a methodological framework for kinesiological analysis using wearable inertial sensor systems and to demonstrate their clinical applicability in evaluating pathological gait in orthopedic patients. Through this approach, we hope to contribute to more personalized, effective, and widely accessible rehabilitation strategies.

## 2. Materials and Methods

### 2.1. Study Population

The study population consisted of patients who sought physiotherapeutic and rehabilitative treatment at University Hospital “Dr. G. Stranski”—Pleven, Bulgaria, following trauma of the hip joint. The included patients were rehabilitated and examined at the Clinic of Physical and Rehabilitation Medicine (CPRM), both in the inpatient rehabilitation department and outpatient diagnostic-consultative center of the hospital. The sample included patients aged over 65 years who had undergone surgical intervention for hip fractures, either with osteosynthesis metallica (OM) (Group A, n = 15) or arthroplasty (Group B, n = 34). All patients used assistive devices, such as underarm crutches, during the rehabilitation period.

### 2.2. Inclusion and Exclusion Criteria

Patients aged 65 years and older who had undergone surgical treatment for hip fractures (osteosynthesis metallica or arthroplasty) and who were referred for physiotherapeutic rehabilitation at the Clinic of Physical and Rehabilitation Medicine were included. Exclusion criteria were presence of neurological disorders affecting gait (e.g., stroke, Parkinson’s disease), severe cognitive impairment limiting compliance with instructions, comorbid musculoskeletal conditions severely affecting mobility (e.g., advanced osteoarthritis in other lower limb joints), or inability to provide informed consent. Patients with controlled chronic conditions such as hypertension or diabetes were included, provided these did not affect gait independently.

### 2.3. Study Design

This observational study employed a “before–after” design, with measurements taken at two time points: at the start of the rehabilitation period (first treatment course with assistive devices) and at the end of the observed period, defined as the time when patients were allowed full weight-bearing on the injured limb. Patients were allowed to bear their full weight on the injured limb at variable times depending on the surgical intervention and surgeon’s protocol, generally ranging from 4 to 8 weeks post operation. The end of the observed rehabilitation period corresponded to the time when full weight-bearing was permitted clinically. The study duration spanned from July 2023 to December 2024. The observed period was shorter than the entire rehabilitation period, focusing specifically on the gait recovery process.

### 2.4. Sample Size Considerations

No a priori power analysis was performed due to the observational nature of the study and limited patient availability during the recruitment period. The sample size was based on clinical feasibility. Results should be interpreted in light of this limitation.

### 2.5. Gait Analysis Methodology

Gait parameters were recorded using the portable inertial sensor device G-WALK (BTS Bioengineering, Milan, Italy). The sensor was positioned at the pelvic region, centered approximately at the level of the fifth lumbar vertebra (L5) or first sacral vertebra (S1), to capture objective functional movement data. This device comprises four integrated sensor modules, each containing a triaxial accelerometer, gyroscope, and magnetometer. Utilizing sensor fusion technology, it combines data from these sensors to enhance the sensitivity and accuracy of motion tracking. Although the G-WALK system incorporates multiple inertial sensors within a single unit (accelerometer, gyroscope, magnetometer), the device operates as an integrated sensor fusion platform. Therefore, spatial and temporal alignment between sensors is managed internally by the device’s firmware and sensor fusion algorithms, ensuring synchronized and accurate measurement outputs without the need for external calibration procedures.

**Figure 2:**
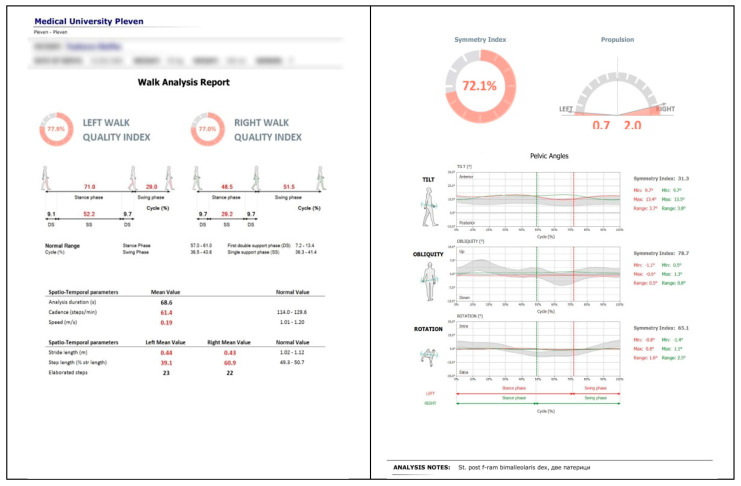
Example of gait analysis output using the BTS G-WALK sensor system (BTS Bioengineering, Italy) in a post-injury patient.

The G-WALK device records various gait parameters including gait speed, cadence, and step length, with the primary focus of this study on pelvic oscillations in the sagittal, frontal, and transverse planes, expressed as percentages relative to normal reference ranges (see Figure 2).

Data are transmitted wirelessly via Bluetooth to dedicated software that processes and computes a comprehensive set of spatiotemporal and kinematic gait metrics. The system has a sampling frequency of 200 Hz for the fused sensor output, ensuring high-resolution motion capture.

Technical specifications include dimensions of 70 × 40 × 18 mm, a weight of 37 g, and an 8 h rechargeable battery life. The inertial sensors support selectable sensitivity ranges (±2 to ±16 g for the accelerometer and ±250 to ±2000 °/s for the gyroscope), while the magnetometer measures magnetic fields up to ±1200 µT. Data are stored internally on a 256 MB flash memory, allowing continuous recording during gait trials.

The G-WALK system has been validated in previous clinical studies involving populations with musculoskeletal and neurological gait impairments, demonstrating good reliability and test–retest reproducibility, as well as agreement with optical motion capture systems for spatiotemporal gait parameters [24]. While specific validation in post-hip surgery elderly patients is limited, the device’s robust sensor fusion algorithms and prior use in orthopedic settings support its applicability in this study.

The gait analysis was performed in a clinical environment within the physical therapy ward of CPRM. Each patient was briefed on the procedure and gave informed consent prior to testing. The examination began with a brief anamnesis and review of medical documentation, followed by anthropometric measurements (including limb circumferences and joint range of motion) recorded in an individual patient file.

Patients were registered in the G-WALK software database with their anthropometric data, age, sex, and diagnosis. The sensor was calibrated via Bluetooth connection to a laptop. The Bluetooth calibration was performed using the proprietary BTS Bioengineering software designed specifically for the G-WALK system. This software ensures reliable data transmission between the sensor and the computer, minimizing data loss or signal interference during gait recording. Patients walked a straight, 10 m corridor at a self-selected comfortable speed, free from obstacles. Data collected during each gait trial were stored within the device software and documented in the individual patient file.

### 2.6. Statistical Analysis

Data were analyzed using SPSS software (IBM SPSS Statistics 28.0). Descriptive statistics including mean, standard deviation (SD), coefficient of variation (Cv%), standard error, and 95% confidence intervals (CIs) were calculated. Comparative analysis of pre- and post-rehabilitation measurements was conducted using paired *t*-tests to assess the significance of changes in gait parameters.

### 2.7. Ethics Approval and Consent to Participate

The research protocol was reviewed and approved by the Ethics Committee of the Medical University, Pleven, in accordance with the ethical principles outlined in the Declaration of Helsinki. The study was conducted at the University Hospital “Dr. G. Stranski”, Pleven, and adhered to the provisions of Ordinance No. 14 of 27 September 2007, which governs the conditions and procedures for conducting therapeutic and non-therapeutic scientific research involving human subjects in Bulgaria.

All participants received detailed information about the purpose and procedures of the study and provided written informed consent prior to enrollment. Consent was obtained using a standardized clinical consent form, ensuring that participants were fully informed about their involvement in the research.

### 2.8. Data Availability

The data supporting the findings of this study are stored securely on the hospital’s computer system and are not publicly available due to privacy and institutional restrictions. Data can be made available from the corresponding author upon reasonable request.

### 2.9. Generative AI Use

Generative artificial intelligence (ChatGPT) (ChatGPT (OpenAI), version GPT-4, accessed in 14 June 2025) was used to assist with language editing and formatting of the manuscript. All scientific content was reviewed and validated by the authors.

## 3. Results

This section presents the outcomes of the rehabilitation process in elderly patients with hip joint trauma, comparing key gait parameters recorded at two time points: first, during ambulation with assistive devices, and second, after transitioning to independent walking without aids. Patients were divided into two groups—those who underwent osteosynthesis (Group A) and those who received hip arthroplasty (Group B). The results focus on pelvic motion in the sagittal, frontal and transverse planes, offering objective insight into postural control and gait recovery throughout the rehabilitation process.

**Figure 3:**
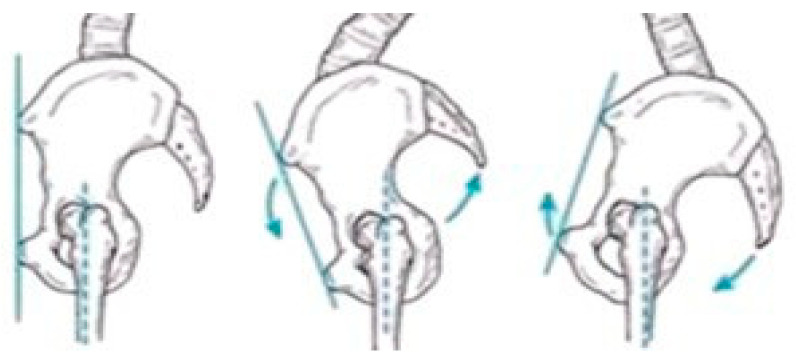
Pelvic oscillation—S plane.

**Figure 4:**
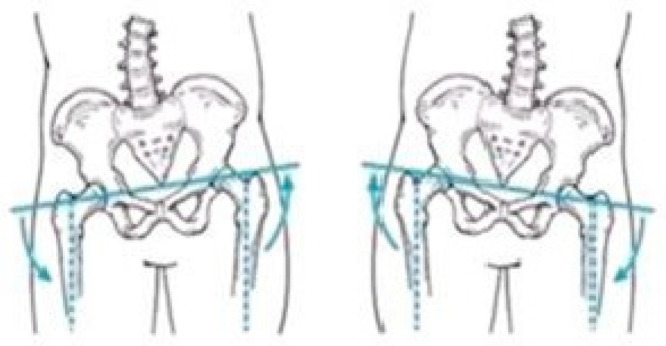
Pelvic oscillation—F plane.

**Figure 5:**
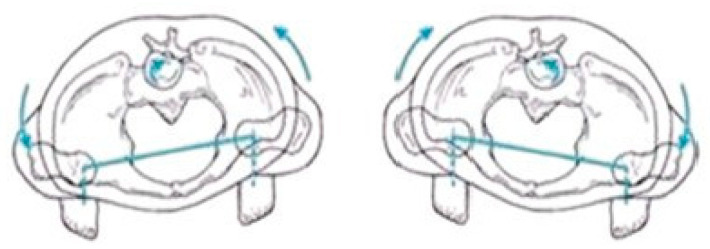
Pelvic oscillation—T plane.

Figure 3, Figure 4 and Figure 5 present illustrative diagrams of pelvic kinematics across the three anatomical planes—sagittal (S), frontal (F), and transverse (T)—highlighting the typical directions and amplitudes of pelvic motion observed during walking. These schematic representations serve as visual aids to better comprehend the complex, multidimensional oscillations of the pelvis that are crucial for maintaining balance and efficient gait mechanics. Each diagram emphasizes key movement components such as anterior–posterior tilt in the sagittal plane, lateral tilt in the frontal plane, and rotational motion in the transverse plane. Although schematic and not direct patient recordings, these figures correspond closely to the parameters quantitatively assessed using the G-WALK inertial sensor system in this study. By providing a clear visualization of pelvic movement patterns, these diagrams facilitate interpretation of the gait data and support understanding of the biomechanical adaptations occurring throughout the rehabilitation process.

### 3.1. Analysis of Pelvic Oscillation in the Sagittal Plane

The statistical data on pelvic oscillation in the S plane, expressed as percentages (%), are summarized in Table 1. The normal reference range for this parameter is between 90% and 100%. Values were recorded at two time points—at the beginning and at the end of the study period for both patient groups: Group A (OM) and Group B (arthroplasty).

As shown in Table 1, Group A exhibited a slight increase in pelvic oscillation values in the S plane, rising from 68.8% to 71.7% by the end of the rehabilitation period. In contrast, Group B demonstrated a slight decrease from 76.4% to 69.1%. However, the changes in both groups were not statistically significant (*p* = 0.313 for Group A; *p* = 0.065 for Group B). These findings reflect variable responses to rehabilitation, with Group A showing gradual progress and Group B exhibiting a slight decrease, possibly due to differences in surgical intervention or recovery dynamics. Table 1 shows these trends by comparing average pelvic motion values at both time points. Despite the lack of statistical significance, such changes underscore the importance of continuous monitoring and individualized rehabilitation strategies, particularly in elderly patients recovering from hip trauma. Further investigation with a longer follow-up period may help clarify whether these patterns persist or stabilize over time. It is also possible that external factors such as post-operative pain, muscle weakness, or fear of movement may have influenced pelvic control during walking. Identifying such factors could improve rehabilitation planning and enhance gait restoration outcomes.

### 3.2. Analysis of Pelvic Oscillation in the Frontal Plane

The statistical data on pelvic oscillation in the F plane, expressed as a percentage (%) relative to the normal reference range of 90–100%, are presented in Table 2. These values were recorded at two time points during the rehabilitation period—initially during ambulation with assistive devices and later during independent walking across both study groups.

As shown in the table, both groups demonstrated significant improvement in F plane pelvic motion over the course of rehabilitation. Group A showed a substantial increase from 46.4% at the initial assessment to 75.2% at the final time point (*p* = 0.001), indicating enhanced lateral stability and improved postural control. Similarly, Group B improved from 52.6% to 72.2%, also reaching statistical significance (*p* = 0.001). These results suggest that rehabilitation interventions led to meaningful gains in frontal plane control in both osteosynthesis and arthroplasty patients. Although neither group fully reached the normal reference range lower limit, the progress reflects a substantial reduction in lateral instability during gait. Improvements in this plane are particularly relevant, as lateral pelvic control is crucial for safe ambulation and fall prevention. The findings reinforce the clinical relevance of targeting frontal plane mechanics during rehabilitation, especially in elderly patients recovering from hip trauma. The consistent improvement observed in both groups underscores the effectiveness of tailored rehabilitation programs in restoring pelvic mobility.

### 3.3. Analysis of Pelvic Oscillation in the Transverse Plane

The statistical data on pelvic oscillation in the T plane, expressed as a percentage (%) relative to the normal reference range of 90–100%, are presented in Table 3. These values were recorded at two time points during the rehabilitation period—initially while walking with assistive devices and subsequently during independent walking for both study groups.

Table 3 also presents the mean values of pelvic oscillation in the T plane for both groups at the beginning and end of the rehabilitation period. Group A demonstrated the greatest progression among all planes, with mean oscillation values increasing significantly from 47.7% to 80.2% (*p* = 0.001). Group B showed a moderate increase from 73.0% to 80.5%; however, this change did not reach statistical significance (*p* = 0.068). The pronounced improvement in Group A may be attributed to post-operative pain, which often leads to compensatory pelvic movements as patients adapt their gait to minimize discomfort. In contrast, patients in Group B, who received hip arthroplasty, typically experience less post-operative pain due to the artificial joint, resulting in less initial compensation via pelvic motion. This difference likely explains the higher baseline pelvic oscillation in Group B and the smaller, less significant increase over time.

While statistically significant improvements were observed in pelvic oscillations within the frontal plane for both groups, the clinical significance of these changes requires further evaluation. The increased pelvic stability likely contributes to improved postural control and reduced fall risk during ambulation, which are critical factors in elderly rehabilitation. However, some pelvic oscillation values remained below normative reference ranges, indicating that residual deficits may persist despite rehabilitation. Pain, which was not systematically assessed in this study, may represent an important factor influencing pelvic motion and gait adaptations. Post-operative discomfort can lead to compensatory movement patterns, affecting rehabilitation outcomes and pelvic control. Future studies should incorporate pain evaluation to better interpret the relationship between pain levels and gait recovery. Defining minimal clinically important differences for pelvic kinematics in this population would strengthen the interpretation of these findings and guide therapy optimization.

Overall, the observed changes in pelvic oscillations across the S, F, and T planes reveal meaningful patterns of gait recovery among patients following hip joint surgery. The inertial sensor-based gait analysis system (G-WALK) provided objective, quantitative measures of pelvic kinematics, allowing for precise and non-invasive monitoring of the rehabilitation progress. While statistically significant improvements were most pronounced in the F plane, the data underscore the utility of wearable sensor technology in capturing subtle biomechanical changes during the transition from assisted to independent ambulation. These findings not only advance our understanding of postural control adaptations but also demonstrate the practical applications of portable inertial sensors in clinical rehabilitation settings. The following discussion will contextualize these results within the broader literature and consider implications for patient care and sensor-based assessment methodologies.

## 4. Discussion

This study examined the rehabilitation outcomes of elderly patients with hip joint trauma, employing a wearable inertial sensor system (BTS G-WALK) to objectively assess pelvic kinematics during gait. The use of this portable sensor technology allowed a precise, real-time capture of spatiotemporal and kinematic gait parameters in clinical settings, demonstrating its potential as a valuable tool for monitoring functional recovery in rehabilitation [25].

Our findings showed positive trends in pelvic oscillations across the sagittal, frontal, and transverse planes during the progression from assisted walking with devices to independent ambulation. Significant improvements were particularly evident in the frontal plane for both osteosynthesis (Group A) and arthroplasty (Group B) patients, with Group A also exhibiting notable progression in the transverse plane. These results are consistent with prior studies emphasizing the importance of pelvic control in maintaining gait stability and efficiency following hip surgery [26,27]. The greater improvement observed in Group A’s transverse plane oscillations may reflect compensatory mechanisms associated with post-operative pain and joint preservation, whereas the relatively stable values in Group B align with their use of artificial joints, which tend to reduce pain and mechanical compensation.

The wearable inertial sensor system enabled a detailed analysis of three-dimensional pelvic movements, providing insights that are difficult to achieve with conventional gait analysis methods or subjective clinical evaluation alone. However, it should be noted that the G-WALK system has limited validation specifically in elderly populations undergoing osteosynthesis or arthroplasty, and its sensitivity to detect subtle kinematic differences in these groups requires further investigation. This highlights the growing relevance of wearable sensor technologies in rehabilitation medicine, as they facilitate objective quantification of complex movement patterns and enable continuous monitoring in diverse settings—ranging from clinical environments to patients’ homes [28,29]. Such capabilities are crucial for tailoring individualized rehabilitation programs and optimizing therapy outcomes.

While the improvements observed in the frontal and transverse planes were statistically significant or approaching significance, changes in the sagittal plane were less pronounced and did not reach significance within the study period. This variability underscores the multifactorial nature of gait recovery, influenced by factors including pain, muscle strength, joint integrity, and patient compliance. However, it is important to note that pain was not systematically evaluated in this study. Post-operative pain can significantly affect gait patterns and pelvic control, potentially leading to compensatory movements that influence rehabilitation outcomes. Future studies should incorporate standardized pain assessments to better elucidate the relationship between pain levels and gait recovery, enabling more tailored and effective rehabilitation strategies. It also suggests that pelvic motion in the sagittal plane may require longer rehabilitation or additional therapeutic focus to achieve significant gains. While some improvements have reached statistical significance, their clinical significance remains uncertain. Establishing minimal clinically important differences for pelvic oscillation metrics would provide clearer interpretation. Nevertheless, the improvements observed in the frontal plane may reflect meaningful gains in balance and lateral stability—key factors in reducing fall risk among elderly patients.

The integration of wearable sensors like the BTS G-WALK within rehabilitation protocols offers several advantages: non-invasiveness, portability, ease of use, and the ability to generate automated, quantitative reports. These features support clinicians in making informed decisions and tracking patient progress objectively over time. Moreover, the adoption of wearable sensor technology aligns with emerging trends in digital health and tele-rehabilitation, which have gained increasing importance, especially in contexts limiting frequent clinical visits [30].

A key limitation of this study is the relatively small sample size and the absence of an a priori power analysis, which may have limited the ability to detect statistically significant changes in some gait parameters, particularly in the sagittal plane. Future studies with larger cohorts are needed to confirm these findings and assess their generalizability.

Future research should investigate the longitudinal application of wearable sensor monitoring over extended rehabilitation periods and in larger patient populations. Incorporating machine learning algorithms could further enhance data interpretation, enabling predictive modeling and real-time adaptive feedback for patients. Additionally, correlating sensor-derived gait parameters with patient-reported outcome measures would provide a holistic view of recovery, integrating objective data with subjective experience.

In summary, this study demonstrates that wearable inertial sensors provide a robust and practical approach for assessing gait and pelvic kinematics during rehabilitation following hip trauma. By facilitating detailed, objective movement analysis, these technologies have the potential to revolutionize rehabilitation assessment and contribute to more effective, personalized treatment strategies in musculoskeletal care.

## 5. Conclusions

This study demonstrated the valuable application of wearable inertial sensor technology in objectively assessing pelvic kinematics during gait rehabilitation in elderly patients following hip surgery. The use of the G-WALK sensor system enabled precise quantification of pelvic oscillations in the sagittal, frontal, and transverse planes at two critical time points—ambulation with assistive devices and independent walking without aids. Significant improvements in pelvic motion were observed, particularly in the frontal and transverse planes, reflecting enhanced postural control and gait recovery. Although changes in the sagittal plane were less pronounced, the overall trends suggest positive functional gains.

The portability, ease of use, and real-time data acquisition capabilities of the wearable sensor provided a comprehensive and clinically relevant evaluation that traditional gait assessment methods may lack. This technology facilitated detailed monitoring of subtle gait compensations and rehabilitation progress in a non-invasive, repeatable manner across clinical and ambulatory settings.

These findings underscore the potential of wearable inertial sensors to enhance individualized rehabilitation protocols by enabling objective, continuous, and quantitative gait analysis. Future research should focus on expanding the sample size, exploring long-term outcomes, and integrating sensor data with machine learning algorithms to further optimize patient-specific rehabilitation strategies. The integration of such advanced sensor technology into routine clinical practice holds promise for improving functional recovery and quality of life in patients with lower limb musculoskeletal impairments.

## Figures and Tables

**Table 1:** Pelvic oscillation in the sagittal plane (%): comparative values at initial and final time points for Groups A and B.

Indicator	n	At the Beginning				In the End				*t*-Test	*p*
		$\bar{X}$	SD	Cv%	CI 95%	$\bar{X}$	SD	Cv%	CI 95%		
Group A	15	68.8	26,606	38.7	49.1 ÷ 78.6	71.7	12,852	17.9	64.6 ÷ 78.8	−1.036	=0.313
Group B	34	76.4	17,888	23.4	70.2 ÷ 82.7	69.1	13,927	20.2	64.3 ÷ 74.0	1.878	=0.065

**Table 2:** Pelvic oscillation in the frontal plane (%): comparative values at initial and final time points for Groups A and B.

Indicator	n	At the Beginning				In the End				*t*-Test	*p*
		$\bar{X}$	SD	Cv%	CI 95%	$\bar{X}$	SD	Cv%	CI 95%		
Group A	15	46.4	27,669	59.6	31.1 ÷ 61.7	75.2	14,407	19.2	67.2 ÷ 83.1	−3.576	=0.001
Group B	34	52.6	26,263	49.9	43.4 ÷ 61.7	72.2	18,027	25.0	65.9 ÷ 78.5	−3.588	=0.001

**Table 3:** Pelvic oscillation in the transverse plane (%): comparative values at initial and final time points for Groups A and B.

Indicator	n	At the Beginning				In the End				*t*-Test	*p*
		$\bar{X}$	SD	Cv%	CI 95%	$\bar{X}$	SD	Cv%	CI 95%		
Group A	15	47.7	29,960	62.8	31.1 ÷ 64.3	80.2	11,826	14.7	73.6 ÷ 86.7	−3.908	= 0.001
Group B	34	73.0	18,802	25.8	66.5 ÷ 79.6	80.5	14,183	17.6	75.6 ÷ 85.5	−1.857	= 0.068

## Data Availability

The data presented in the study are available on request from the corresponding author. The data are not publicly available due to privacy and ethical restrictions.

## Abbreviations

The following abbreviations are used in this manuscript:

COM	Center of mass
CPRM	Clinic of Physical and Rehabilitation Medicine
EMG	Electromyography
F	Frontal
FS	Floor sensor
IP	Image processing
IMUs	Inertial measurement units
LRS	Laser range scanners
NWS	Non-wearable sensor
OM	Osteosynthesis metallica
S	Sagittal
T	Transverse
ToF	Time-of-flight
WS	Wearable sensor
